# Reproducibility in animal models of hypertension: a difficult problem

**DOI:** 10.1186/s13293-018-0216-4

**Published:** 2018-12-22

**Authors:** Jane F. Reckelhoff, Barbara T. Alexander

**Affiliations:** 1 Department of Cell and Molecular Biology, University of Mississippi Medical Center, Jackson, USA; 2 Department of Physiology, University of Mississippi Medical Center, Jackson, MS 39216-4505 USA; 3 Mississippi Center of Excellence in Perinatal Research, University of Mississippi Medical Center, Jackson, USA; 4 Women’s Health Research Center, University of Mississippi Medical Center, Jackson, MS 39216-4505 USA

**Keywords:** Dahl salt-sensitive rat, Spontaneously hypertensive rat, Rag-1 knockout mouse

## Abstract

In 2016, the National Institutes of Health mandated that all grant proposals enhance reproducibility through rigor and transparency. In the past few years, physiological outcomes in established animal models of hypertension, in particular in regard to sex differences, have varied from study to study or laboratory to laboratory. The aim of this commentary is to increase investigator awareness of caveats related to animal models that may be sensitive to vendor-, barrier-, or diet-specific changes that result in an inability to sustain the genotype and/or phenotype of well-established experimental models. These considerations are critical in order for investigators to make informed and educated decisions in regard to their hypothesis-driven research, in particular as it relates to experimental design and interpretation, and the reporting of results.

In January 2016, the National Institutes of Health (NIH) mandate for Rigor and Reproducibility took effect for all research grants and mentored career development award applications. These guidelines were based on data presented in 2014 at a conference at the NIH that was jointly sponsored by *Nature*/*Science* journals on the issue of reproducibility and rigor in preclinical research findings. As a result, many journals and journal editors also signed off on the guidelines to support preclinical research that is “reproducible, robust, and transparent.”. However, in recent years, animal models that have been historically used for the study of the mechanisms responsible for hypertension have undergone changes in genotype and/or phenotype compromising the ability of investigators to maintain reproducibility.

For example, in 2016, Zimmerman and Lindsey pointed out in a Letter to the Editor of the *American Journal of Physiology Renal Physiology* a shift in blood pressure in female Dahl salt-sensitive (DS) rats kept on a low-salt diet [1]. Their observations included a study by Gillis et al. published in 2015 that reported female DS rats descended from the original Rapp strain maintained at the University of Mississippi Medical Center and fed a 0.3% NaCl phytoestrogen replete diet had a mean arterial pressure (MAP) of 155 mmHg at approximately 17 weeks of age [2]. MAP in this study was measured by radiotelemetry, the gold standard for recording of arterial pressure in rodents [3]. Yet, Brinson and colleagues reported that MAP was 125 mmHg at 16 weeks of age in DS females obtained from the breeding colony at Georgia Regents, now Augusta University, and fed a 0.4% NaCl phytoestrogen-free diet [4]. Blood pressure in this study was also measured via telemetry [4]. Zimmerman and Lindsey also included studies from their own laboratory demonstrating DS rats from Harlan (now Envigo) or Charles River placed on a 0.3% NaCl phytoestrogen replete diet, or fed a 0.3% or 0.5% NaCl phytoestrogen free diet, exhibited a significant difference in systolic blood pressure (SBP) at 15 to 16 weeks of age measured by tail cuff in the conscious state, a difference of 200 mmHg versus 155–170, respectively [1]. These differences in blood pressure in female DS rats occurred despite the potential for a phytoestrogen-free diet to increase BP compared to a diet containing phytoestrogens, although this effect could be sex and age-dependent [5, 6]. Our laboratory reported in 2016 that MAP in 16-week-old DS female rats obtained from Harlan (now Envigo) and fed a 0.3% NaCl phytoestrogen replete diet was also 155 mmHg [7], whereas MAP in a study from our laboratory in 2010 was only 105 mm Hg [8]. Both studies were performed in female DS rats obtained from the same vendor; however, the study published in 2010 was performed in female rats at 10 weeks of age that were fed a 0.3% phytoestrogen, nitrate/nitrite-free diet. In our 2010 study, MAP was similar in intact female DS relative to ovariectomized (OVX) DS maintained on a low-salt diet [8]. However, when placed on a high salt diet (8% NaCl), MAP in OVX DS rats was significantly more salt-sensitive (i.e., increased to a greater degree) than intact female DS on high salt [8]. Greater salt sensitivity was also reported in OVX DS in the study published by Brinson et al. [4]. However, exposure to a high salt diet (8%) did not further increase SBP in intact or OVX DS rats as reported by Zimmerman and Lindsey [1]. Based on our previous data, and those of many others, one would expect a consistent salt-sensitive increase in blood pressure in OVX DS rats on a high-salt diet. Collectively, based on these observations, Zimmerman and Lindsey concluded that greater transparency was needed from both commercial vendors and from investigators. We agree and conclude that the published differences certainly suggest there may be genomic differences impacting the phenotype drifts in blood pressure in the DS rat strain. It is important to note that as far back as 2005, Mattson et al. demonstrated that the blood pressure response in sodium-dependent hypertension in the DS rat is nutrient specific with the dietary source of protein playing an important role in the development of hypertension in the DS rat [9]. Clearly, this study from the Mattson laboratory highlights the importance of biological variables including dietary components on physiological outcomes.

In 2018, Pai and colleagues reported that DS females exhibit spontaneous hypertension when maintained on a low-salt (0.1%), phytoestrogen replete (Teklad) diet [10]. In addition, OVX at 3 months of age had no effect on MAP or body weight by 7 months of age, and MAP averaged 160–163 mmHg in intact and DS rats, respectively [10]. They also found that OVX at 6 weeks of age (prepubertal) had no effect on MAP by 10 months of age, compared to intact females [10]. These investigators went on to compare the genetics of the SS/JrHsd/Env (the Envigo DS rats) with the SS/Jr (the original Rapp strain maintained at University of Toledo) and the SS/JrHsd/Mcwi strain maintained at the Medical College of Wisconsin [10]. While complete direct genomic comparisons were not feasible, they reported more single nucleotide polymorphisms and insertion-deletion polymorphisms in SS/JrHsd/Env than in the SS/JrHsd/Mcwi and SS/Jr. They also showed more genetic variants when comparing the SS/JrHsd/Env than in the other two strains. In addition, SS/JrHsd/Env had a greater number of heterozygous/homozygous ratios than the other two strains [10].

The Rag1^−/−^ mouse on a C57Bl/6 J background is another example of divergence in observed physiological outcomes. The Rag1^−/−^ mouse is T cell deficient due to a targeted mutation of the recombination-activating gene-1 (Rag1) [11]. In 2007, Guzik and colleagues reported that Rag1^−/−^ mice had no pressor response (no increase in blood pressure) to high doses of angiotensin (Ang) II [12]. Over the subsequent years, studies similar to these, using similar doses of Ang II, were repeated by four additional laboratories with similar results [13–16]. However, in 2017, Ji and colleagues reported that MAP in the Rag1^−/−^ mice was no longer refractory to Ang II [17] and that the pressor response to even a low dose of Ang II, 490 ng/kg, was similar between the Rag1^−/−^ mice and wild-type mice. The subsequent increase in MAP in response to Ang II in Rag1^−/−^ was shown to be independent of T cells in this study [17].

In 2014, Taconic Laboratories stopped providing the spontaneously hypertensive rats (SHR). Prior to this time, we noticed that one or both kidneys of approximately 40% of both male and female SHR exhibited increased hydronephrosis, often with little medulla present (unpublished findings). We contacted the company with pictures of the kidneys and subsequently moved away from using these animals. This was difficult for us since we had been using this strain and vendor source for these animals to study sex differences in blood pressure regulation both in young adult animals and in rats aged up to 20 months, since the mid-late 1990s, and had more than 40 publications with them. In order to continue studies in this rat stain with another vendor, we are repeating even the simplest studies, such as the effect of sex steroids on blood pressure, to validate our earlier findings. It is important to note that the SHR is a commonly used animal model of essential hypertension. While we reported that antioxidants like tempol reduce MAP in male SHR, but have little effect in female SHR at 12 weeks of age [18], a new study by Gillis and colleagues using the Envigo SHR showed no effect of tempol on MAP in male or female SHR at 12 weeks of age when measured with telemetry [19]. These findings are also contrary to studies by others that report a significant reduction in MAP in tempol-treated male SHR [20, 21].

Taken together, these studies indicate that while the ability to reproduce studies of other investigators, or studies performed even within our own laboratories, is an important and laudable goal, it may be hampered from year to year due to diverging genomics that affect the phenotype of the experimental animal models. This is especially important for the study of sex differences in biological systems. For example, we now know that diet plays a huge role in mediating sex differences, since new data show that the microbiome affects the levels of sex steroids [22]; thus, sex steroid-mediated responses can change with different diets. Vendors must be cognizant of this and remain transparent in their reporting of change in diets to investigators that utilize these experimental rodent strains. Investigators should also be diligent and aware of the effect that relevant biological variables such as diet, vendor, or source including changes in commercial barrier may have on their predicted or expected outcomes.

Thus, the recent mandate by NIH in regard to consideration of relevant biological variables is timely. Consideration of relevant biological variables is critical in order to maintain rigor and reproducibility in animal studies and resides not only with the investigator, but also with the vendors who provide the experimental animal models and rodent strains used by numerous scientists. In a perfect world, vendors are expected to maintain the quality of their rodent strains. They should hold to a high level of transparency by providing information on their websites any time they make *any* changes that could impact the genotype/phenotype of their animals. Maintenance of these experimental models and rodent strains should also include routine genotyping. Vendors would likely argue that this would be prohibitively expensive and thus not feasible. In addition, most vendors change husbandry, breeding, feeding, and maintenance protocols to become more cost-efficient. Unfortunately, they do so in most cases without informing the investigators who spend large sums of money and depend on these animals to remain consistent over long periods of time. Having investigators go to the expense and work of maintaining their own colonies is also not the solution since it defeats the purpose of having commercial vendors to supply large numbers of animals that are consistent as possible in their genotype and phenotype in order to reinforce reproducibility of scientific findings. It is also imperative that investigators promote reproducibility by providing as much detail as possible regarding husbandry, diet, and housing conditions of the animals being studied, include explicit information on the methods used and as noted in the mandate by the NIH, and subscribe to a high level of rigor in their experimental design and reporting of results.

This commentary was written to raise awareness regarding the frustration of scientists in their inability to reproduce blood pressure data in commonly used experimental animal models and the subsequent studies some investigators have performed to investigate why this is occurring. This commentary should serve as an alert to others that their experimental animal models or systems may be changing with time. We provide no solutions to the problem of changing genotypes/phenotypes in the models we typically use, but believe that rigor and reproducibility is still the ultimate goal. Investigators need to determine what animal model will best allow them to address their hypotheses, with the caveat that some of their results may not be exactly as they or others have previously published. Publication of these differences is imperative in order to ensure the ongoing documentation of the effect of biological variables on experimental outcomes.
